# Supplementary material for: Deciphering genomic codes using advanced NLP techniques: a scoping review
Source: ArXiv. 2024 Nov 25:arXiv:2411.16084v1. Preprint. [Version 1] (PMC11623714)
Supplement: Supplement 1 [file NIHPP2411.16084v1-supplement-1.pdf]

A PRISMA checklist

This review adheres to PRISMA guidelines to ensure methodological rigor and transparency. Key aspects of the PRISMA checklist addressed in this review include defining clear objectives and eligibility criteria, describing information sources and search strategies, and outlining the study selection and data collection processes. Additionally, we summarize study characteristics, assess the risk of bias, and present the synthesis results and findings. Limitations are discussed, along with an assessment of evidence certainty to contextualize the main findings. Funding sources and conflicts of interest are also disclosed to maintain transparency.

For further details on the PRISMA checklist and specific reporting requirements, please refer to the full PRISMA 2020 checklist available at `http://www.prisma-statement.org/PRISMAStatement/Checklist`.

B Search Strategies

Medline (04/12/24)

1. exp Natural Language Processing/
2. exp Data Mining/
3. ((natural adj2 language adj2 process*) OR (large adj2 language adj2 model*) OR ”NLP” OR ”LLM” OR ((data OR text) adj2 (mine* OR ”mining”)) OR ”named entity” OR semantic).ti,ab.
4. 1 OR 2 OR 3
5. exp Genetic Association Studies/
6. exp Polymorphism, Genetic/
7. (genet* OR genom* OR genot* OR sequenc* OR ((single adj2 nucleotide adj2 polymorph*) OR ”SNP*”)).ti,ab.
8. 5 OR 6 OR 7
9. (token* OR ”transformer” OR ”bert” OR (regulat* adj2 annotat*)).ti,ab.
10. 4 AND 8 AND 9

Embase (04/12/24)

1. exp natural language processing/
2. exp data mining/
3. ((natural adj2 language adj2 process*) OR (large adj2 language adj2 model*) OR ”NLP” OR ”LLM” OR ((data OR text) adj2 (mine* OR ”mining”)) OR ”named entity” OR semantic).ti,ab.
4. 1 OR 2 OR 3
5. exp genetic association study/
6. exp genetic polymorphism/
7. (genet* OR genom* OR genot* OR sequenc* OR ((single adj2 nucleotide adj2 polymorph*) OR ”SNP*”)).ti,ab.
8. 5 OR 6 OR 7
9. (token* OR ”transformer” OR ”bert” OR (regulat* adj2 annotat*)).ti,ab.
10. 4 AND 8 AND 9

PubMed (04/12/24)

(”Natural Language Processing”[Mesh] OR ”Data Mining”[Mesh] OR NLP OR LLM OR ”Natural Language Processing” OR ”data mining”)
AND (”Genetic Association Studies”[Mesh] OR ”Polymorphism, Genetic”[Mesh] OR genet* OR genom* OR genot* OR sequenc* OR ”SNP” OR ”SNPs”)

AND (token* OR ”transformer” OR ”bert” OR ”regulatory annotations”)

Scopus (04/15/24)

(TITLE-ABS-KEY ((genet* OR genom* OR genot* OR sequenc* OR ((single W/2 nucleotide W/2 polymorph*) OR ”SNP*”))))
AND (TITLE-ABS-KEY (((natural W/2 language W/2 process*) OR (large W/2 language W/2 model*) OR ”NLP” OR ”LLM” OR ((data OR text) W/2 (mine* OR ”mining”)) OR ”named entity” OR semantic)))
AND (TITLE-ABS-KEY ((token* OR ”transformer” OR ”bert” OR (regulat* W/2 annotat*))))

Web of Science (04/15/24)

((natural NEAR/2 language NEAR/2 process*) OR (large NEAR/2 language NEAR/2 model*) OR ”NLP” OR ”LLM” OR ((data OR text) NEAR/2 (mine* OR ”mining”)) OR ”named entity” OR semantic) (Abstract)
AND (genet* OR genom* OR genot* OR ”sequencing” OR ((single NEAR/2 nucleotide NEAR/2 polymorph*) OR ”SNP*”)) (Abstract)
AND (token* OR ”transformer” OR ”bert” OR (regulat* NEAR/2 annotat*)) (Abstract)

ACM (04/15/24)

[[Abstract: ”natural language processing”] OR [Abstract: or] OR [Abstract: ”large language model”] OR [Abstract: or ”nlp” or ”llm” or] OR [Abstract: ”data mining”] OR [Abstract: ”text mining”] OR [Abstract: ”named entity”] OR [Abstract: or semantic]]
AND [[Abstract: genet* OR genom* OR genot* OR] OR [Abstract: ”single nucleotide polymorph”] OR [Abstract: or ”snp” or ”snps”]]
AND [Abstract: token* OR ”transformer” OR ”bert”]
